# NPS-1034 Induce Cell Death with Suppression of TNFR1/NF-κB Signaling in Testicular Cancer

**DOI:** 10.3390/medicina58030355

**Published:** 2022-02-27

**Authors:** Jian-Ting Chen, Shao-Chuan Wang, Brian-Shiian Chen, Ya-Chuan Chang, Chia-Ying Yu, Wen-Wei Sung, Tuzz-Ying Song

**Affiliations:** 1 Division of Urology, Department of Surgery, Yuanlin Christian Hospital, Changhua 51052, Taiwan; wasiatin@gmail.com; 2 Department of Food Science and Biotechnology, Da-Yeh University, Changhua 51500, Taiwan; 3 School of Medicine, Chung Shan Medical University, Taichung 40201, Taiwan; rosenbeck.wang@gmail.com (S.-C.W.); 1638faang@gmail.com (B.-S.C.); raptor7037@gmail.com (Y.-C.C.); cyyu2015@gmail.com (C.-Y.Y.); 4 Department of Urology, Chung Shan Medical University Hospital, Taichung 40201, Taiwan; 5 Institute of Medicine, Chung Shan Medical University, Taichung 40201, Taiwan

**Keywords:** NPS-1034, TNF receptor-1, p65, p50, apoptosis, testicular cancer

## Abstract

*Background and objectives:* NPS-1034 with a dual inhibitory effect on Met and Axl kinase receptors has exhibited therapeutic potential in previous models. However, no study on treating testicular cancer (TC) cell lines with NPS-1034 has been established. *Materials and Methods:* In this study, a series of in vitro examinations of the apoptotic effect induced by NPS-1034 in TC cell lines was conducted to clarify the molecular interactions involved. *Results:* A decrease in cell viability rate was observed following NPS-1034 treatment, as shown in the MTT assay. Induction of the apoptotic effect was observed in TC cells as the sub-G1 and Annexin-PI populations increased in a dose-dependent manner. The involvement of the tumor receptor necrosis factor receptor 1 (TNFR1) pathway was later determined by the proteome array and western blotting. A reduction in TNFR1 and NF-κB downstream protein expressions, an upregulation of cleaved caspase-3 and -7, and a downregulation of survivin and claspin all reassured the underlying mechanism of the TNFR1 involved in the apoptotic pathway induced by NPS-1034. *Conclusions:* Our findings provide evidence for a potential underlying TNFR1 pathway involved in NPS-1034 treatment. This study should offer new insights into targeted therapy for TC.

## 1. Introduction

Testicular cancer (TC) is a malignant solid tumor that usually affects males, from adolescents to those of middle age [1]. TC is a unique disease due to its high occurrence rate in the young population and heterogeneity of tumor cells [2]. Over the past few decades, the incidence of TC has increased worldwide for unknown reasons [3,4]. The risk factors identified are occupational exposure, environmental exposure, genetic contributions, cryptorchidism, etc. [2,5,6,7]. TC can be divided into two categories based on its neoplasm: testicular germ-cell tumors (TGCT) and sex cord-stromal tumors. TGCT accounts for approximately 95% of all cases, which can be further classified into seminoma and non-seminoma, depending on the original lesion [8]. Current clinical treatments include radiotherapy, chemotherapy, and orchidectomy. A good prognosis as well as a positive response to clinical approaches can be observed in the early stage of TC tumor development [1,9]. However, the aforementioned therapies may affect spermatogenesis and physical function, suggesting the need for further research.

NPS-1034 is an inhibitor of multiple kinases, including Met, Axl, etc. [10]. Met and Axl are aberrantly expressed oncogenes in many malignant tumors [11,12]. Amplified Met receptors are linked to cell proliferation, metastasis, and even invasion, while overexpression of the Axl gene is related to EMT, cell survival, angiogenesis, and resistance to treatment [13,14,15]. NPS-1034 has been proven to be effective in previous studies. For example, it exhibited potency in downregulating the phosphorylation of Met and its downstream signals: Akt and Erk [16]. It also inhibited vascularization in xenograft mice with human gastric cancer cells, and no weight loss was observed in the model [16]. Moreover, NPS-1034 resolved drug resistance caused by amplified Met and Axl gene expression in cancer cell lines [10], synergistically enhanced the efficacy of preexisting epidermal growth factor receptor tyrosine kinase inhibitor (e.g., Erlotinib and Gefitinib), downregulated oncogenic protein expressions, and promoted cell death [10]. Overall, previous studies have presented the great potential of NPS-1034 in inhibiting various signal pathways related to tumor cell survival. However, few articles regarding NPS-1034 have been published, supporting the need for further studies on NPS-1034 for future application.

Although current therapies have exhibited excellent outcomes in general, further therapeutic strategies are required to resolve clinical concerns, such as toxicity and resistance to platinum drugs, post-radiotherapy, and post-chemotherapy cardiovascular impact [17,18,19,20]. To date, no studies have elaborated on NPS-1034 in treating TC. Expression of the Axl gene can be detected in the Sertoli cell, which has been proven to be involved in spermatogenesis in the testis [21]. An aberrant expression of Met receptors was also discovered in TGCT cell lines, both seminoma and non-seminoma, suggesting that the Met gene may be a pathological marker and treatment target for medical development [22]. In summary, we hypothesize that the targeted therapy NPS-1034 may serve as an alternative regimen for inhibiting testicular tumor growth and progression.

## 2. Materials and Methods

### 2.1. Cell Culture

The human TC cell lines NCCIT and NTERA2 were used in the present study. Cells were purchased from the Bioresource Collection and Research Center (Hsinchu, Taiwan). The NCCIT cell line was maintained in RPMI-1640 medium, while the NTERA2 cell line was maintained in Dulbecco’s Modified Eagle medium. Both mediums were supplemented with 10% fetal bovine serum, 100 U/mL penicillin, 100 μg/mL streptomycin, 2 g/mL NaHCO_3_, 1 mM sodium pyruvate, and 0.1 mM non-essential amino acids. All cell lines were kept in a 37 °C incubator at 5% CO_2_.

### 2.2. MTT Assay

Cells were seeded in 96-well plates at a concentration of 10^4^ cells per well and then incubated overnight to allow for cell attachment. The following day, different concentrations of NPS-1034 (MedChemExpress, Monmouth Junction, NJ, USA) were added to the wells, and cell viability was measured at 24 h post-treatment. The MTT reagent was added at a concentration of 0.5 mg/mL in each well and incubated for 3 h. Then, 100 μL of DMSO was added to solubilize the formazan crystals formed, and the sample was read using an ELISA reader at a wavelength of 570 nm as described before [23].

### 2.3. Cell Cycle Analysis

The cells were seeded in 6-well culture plates at a concentration of 3~4 × 10^5^ cells/well and incubated overnight. NPS-1034 were added at 0, 10, and 40 μM and treated for 48 h. After treatment, the cells were collected, and the resulting pellet was fixed in 70% ethanol and stored at −20 °C overnight. To analyze cell cycle phase distribution, cells were re-suspended in PBS containing 4 µg/mL PI and 0.5 mg/mL RNase, and cells were evaluated by flow cytometer afterward. FlowJo software (FACSCanto II; BD Biosciences, San Jose, CA, USA) was used for formal analyses as described before [23]. All samples were tested in triplicate.

### 2.4. Hoechst 33342 Staining

Hoechst 33342 staining was conducted to detect cell apoptosis by cell morphology transformation. Cells were seeded in a 6-well plate and treated with NPS-1034 at 0, 10, and 40 μM for 24 h and harvested at 48 h. Cells were incubated with Hoechst 33,342 (10 μg/mL, Invitrogen) for 20 min after the PBS was properly washed. Subsequently, images were recorded with a fluorescence microscope (ImageXpress PICO, excitation wavelength of 350–390 nm, emission wavelength of 420–480 nm) at 20× magnification. Apoptotic cells emitted blue fluorescence and exhibited morphological changes in the nuclei typical of apoptosis. For quantification of apoptotic cells, five random visual fields were selected to calculate the percentage of apoptotic cells.

### 2.5. The Annexin-V/FITC Assay

An Annexin V-FITC apoptosis detection kit (Elabscience, Houston, TX, USA) was used based on the manufacturer’s protocol. The cells were seeded in 6-well culture plates at a concentration of 3–4 × 10^5^ cells/well and incubated overnight. NPS-1034 was added at 0, 10, and 40 μM and treated for 48 h. Treated cells were harvested, and the resulting pellets were resuspended in 100 ul of binding buffer and subsequently stained with 2 μL Annexin V-FITC and 2 μL PI. After incubating at room temperature for 15 min, the number of apoptotic cells was evaluated by flow cytometry (FACSCanto™ II Cell Analyzer; BD Biosciences, San Jose, CA, USA). For detection of the cell apoptotic rate, FlowJo software (BD Biosciences, San Jose, CA, USA) was used for formal analyses as described before [23]. All tests were performed in triplicate.

### 2.6. The Human Apoptosis Array for Proteome Profiling

The Human Apoptosis Proteome Profiler™ array (R&D Systems, Minneapolis, MN, USA), which is composed of a nitrocellulose membrane duplicate spot of 35 apoptosis-related proteins, was used in the present study. Total protein from NCCIT and NTERA2 cells treated with NPS-1034 0 and 40 μM for 24 h were extracted, and 400 μg of total protein were used for each array, per the manufacturer’s protocol. Each experiment was performed in duplicate, and the integrated density of the spots was quantitated by Image J software (National Institutes of Health, Bethesda, MD, USA).

### 2.7. Western Blotting

Total cell extracts were prepared with RIPA buffer, containing protease inhibitor and phosphatase inhibitor, and centrifuged at 10,400× rpm for 20 min to remove cellular debris. Next, 15 µg of total protein were subjected to gel electrophoresis and transferred to PVDF membranes (Millipore, Burlington, MA, USA). After blocking in 5% non-fat milk for 2 h, the membranes were incubated overnight at 4 °C, with primary antibodies. The next day, the membranes were incubated for 1 h at room temperature with a 5000-fold diluted secondary antibody. Proteins were visualized with the ImmobilonTM-Western Chemiluminescent HRP Substrate (Millipore, Burlington, MA, USA), and the results were shown on the GE Healthcare ImageQuant LAS4000 (Chicago, IL, USA).

### 2.8. Statistical Analysis

Statistical analysis was conducted using IBM SPSS software version 20.0 (Chicago, IL, USA). All data were presented with statistical mean ± standard deviation (SD) from three independent experiments. The student’s *t*-test was used for continuous or discrete data analysis. All statistical tests were two-sided, and values of *p* < 0.05 were considered statistically significant (* *p* < 0.05; ** *p* < 0.01; *** *p* < 0.001).

## 3. Results

### 3.1. NPS-1034 Inhibits the Cell Progression of NCCIT and NTERA2 Cell Lines In Vitro

**Figure 1:**
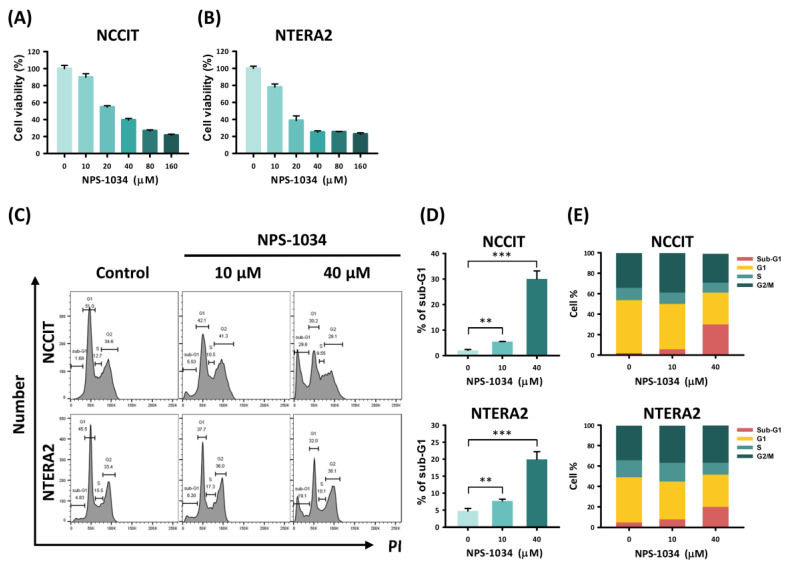
Analysis of TC cell survival with NPS-1034 treatment. The MTT assay was used to determine TC cell viability in (**A**) NCCIT and (**B**) NTERA2 cell lines treated with NPS-1034 (10, 20, 40, 80, and 160 µM) and DMSO (control) for 24 h. (**C**) Cell cycle analysis was performed to evaluate changes in each phase of NCCIT and NTERA2 cell lines treated with NPS-1034 at 10 and 40 µM for 48 h. (**D**) An increase in the sub-G1 phase can be observed in both cell lines after NPS-1034 treatment. (**E**) The proportion of the cell cycle was quantified in the segments of the bar chart. The data are presented as mean ± SD (** *p* < 0.01; *** *p* < 0.001).

To evaluate the potency of NPS-1034 in inhibiting tumor cell growth, we performed a cell viability assay on the TC cells NCCIT and NTERA2. The cells were treated with NPS-1034 at different concentrations (10, 20, 40, 80, and 60 µM) or DMSO (control group) for 24 h. Our results found a dose-dependent viability rate in NCCIT and NTERA2 cells with NPS-1034 treatment, suggesting its inhibitory and cytotoxic effect in vitro (Figure 1A,B). Then, we evaluated the cause of cell death and cell cycle progression. Flow cytometry was used to analyze the proportion of each phase in cancer cells after being treated with NPS-1034 at 10 and 40 µM for 48 h (Figure 1C–E). The result of the NCCIT cell line presented a significant rise in the sub-G1 phase in a dose-dependent manner, from 2.08 ± 0.35% to 30.07 ± 3.13% (NCCIT: control vs. 10 and 40 µM, *p* = 0.003 and 0.001, respectively) (Figure 1D). A similar trend of sub-G1 phase was seen in the NTERA2 cell line at the same drug concentration (Figure 1D), from 4.75 ± 0.74% to 19.93 ± 2.27% (NTERA2: control vs. 10 and 40 µM, *p* = 0.005 and 0.001, respectively), suggesting that NPS-1034 can induce cell death via apoptosis and inhibit mitosis. Overall, the analysis revealed the potential of NPS-1034 in upregulating apoptotic pathways in TC cells.

### 3.2. NPS-1034 Enhances Apoptosis in NCCIT and NTERA2 Cell Lines

**Figure 2:**
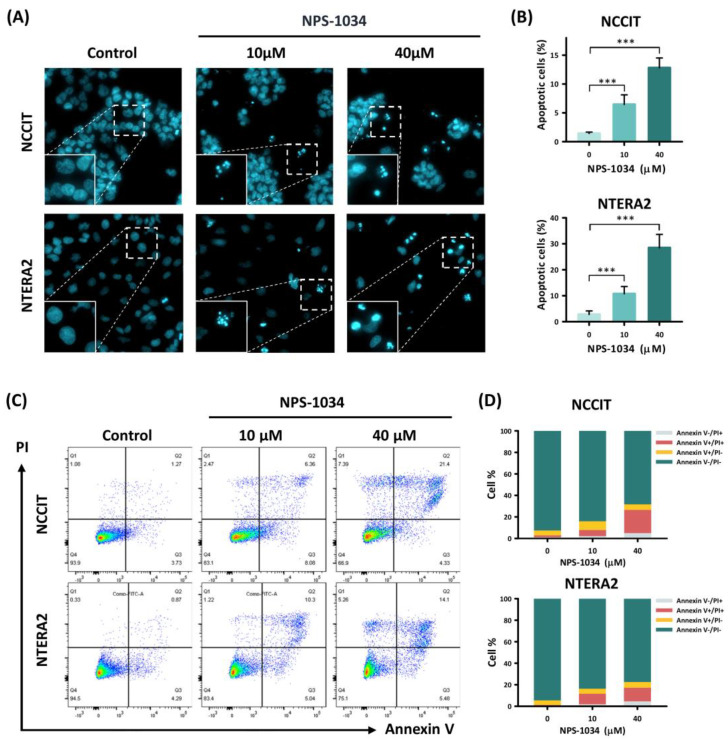
NPS-1034 promotes apoptosis in TC cell lines. (**A**) Hoechst stain 33,342 was used to visualize the apoptotic cells of the NCCIT and NTERA2 cell lines treated with NPS-1034 (0, 10, and 40 µM) for 24 h. (**B**) The percentage of apoptotic cells is presented in the columns. (**C**) Flow cytometry was conducted to evaluate the cause of cell deaths with annexin v/PI dual stain. An examination was performed to determine the number of apoptotic NCCIT and NTERA2 cells treated with NPS-1034 for 48 h. (**D**) The proportion of cell distribution is shown in the column for NCCIT and NTERA2 cells. The data are presented as mean ± SD (*** *p* < 0.001).

To consolidate the hypothesis that NPS-1034 facilitates apoptosis, a fluorescence microscopy analysis was performed with Hoechst stain 33,342. As shown in Figure 2A,B, an increase in intensified blue fluorescence dots can be observed in cells treated with NPS-1034 (10, 40 µM) for 24 h. The bright blue fluorescence dots in Figure 2A visualized the condensed chromatins, suggesting an increase in apoptotic cells. The result of fluorescence microscopy was quantified in a bar chart (Figure 2B). The percentage of apoptotic NCCIT cells treated with NPS-1034 at 40 µM rose from 1.43 ± 0.23% to 12.80 ± 1.71%, compared to the control group (NCCIT: control vs. 10 and 40 µM, *p* = 0.001 and 0.001, respectively), while apoptotic NTERA2 cells escalated from 2.79 ± 1.35% to 28.46 ± 5.51% in groups treated with NPS-1034 at 40 µM (NTERA2: control vs. 10 and 40 µM, *p* = 0.001 and 0.001, respectively). To clarify the cause of cell death, additional flow cytometry analysis was used to evaluate cell death driven by NPS-1034 with Annexin V/PI dual staining (Figure 2C). The sections in the columns in Figure 2D represent the percentage of early and late apoptotic cells after a 48-h NPS-1034 treatment, from 6.27 ± 1.18% to 26.68 ± 5.91% in NCCIT cell lines compared with the control group (NCCIT: control vs. 10 and 40 µM, *p* = 0.001 and 0.004, respectively). As for the NTERA2 cell line, the rate of apoptotic cells rose from 5.15 ± 0.54% to 17.94 ± 1.45% (NTERA2: control vs. 10 and 40 µM, *p* = 0.001 and 0.001, respectively). The accumulation of apoptotic cells at different stages complies with the result in Figure 2A,B, indicating the potency of NPS-1034 in inducing cellular apoptosis in TC.

### 3.3. Identification of Proteins Related to NPS-1034-Facilitated Apoptotic Pathways

**Figure 3:**
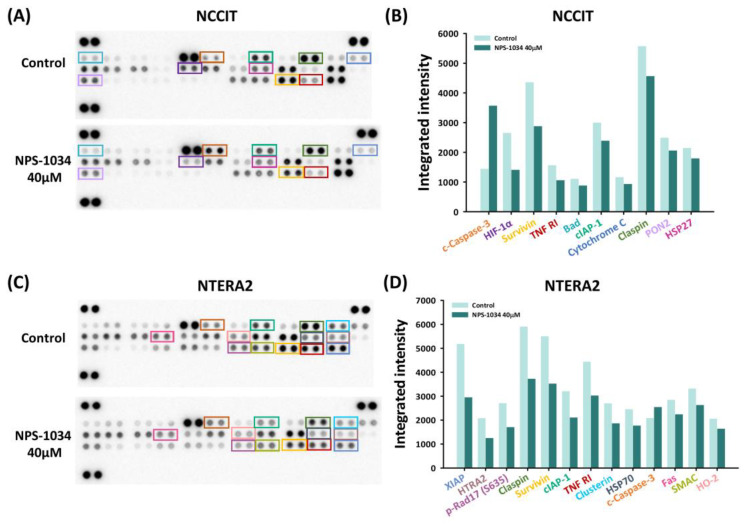
An apoptosis array examined the expressions of proteins involved in cell death caused by NPS-1034. Both (**A**) NCCIT and (**C**) NTERA2 cell lines were tested with an apoptosis array. The expressions of proteins were quantified into bar charts, as shown in (**B**,**D**). The proteins were aligned based on the extent of changes in the protein expressions before and after NPS-1034 treatment.

With preliminary confirmation of the NPS-1034-induced apoptotic effect in TC cell lines, a proteome apoptosis array was conducted to determine the molecular mechanisms involved in NPS-1034-induced pathways. The results shown in Figure 3A–D quantified apoptotic markers with a proteome array. The downregulation of an upstream TNFR1 can be observed in both cell lines. Based on the results (Figure 3B–D), we hypothesize that NPS-1034 regulates the expression of TNFR1 in both NCCIT and NTERA2 cell lines, leading to decreased surviving and claspin expressions, which both possess antiapoptotic effects.

**Figure 4:**
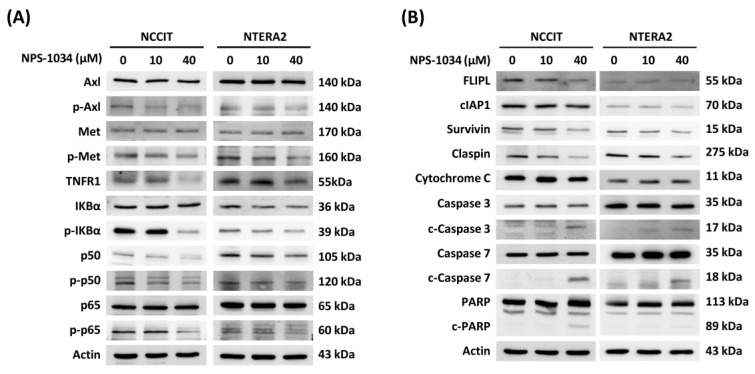
NPS-1034 potentially triggered an apoptotic pathway in TC cell lines via the TNFR1 pathway. (**A**) NPS-1034 inhibits the p-Axl, p-Met and downstream pathway NF-κB of TNFR1, which might lead to certain gene expressions in both NCCIT and NTERA2 cell lines. (**B**) Proteins involved in the apoptotic pathways were determined following the treatment of NPS-1034 in TC cell lines.

A more comprehensive investigation was therefore performed with western blotting to uncover underlying mechanisms of TC cell lines with NPS-1034. Lowered phosphorylated rate in Axl and Met expressions, the two chief targets of NPS-1034, could be observed in our study (Figure 4A). Notably, a reduction in the expression of phosphorylated IKBα and phosphorylated p50 and p65 proteins was presented, suggesting the inhibition of TNFR1/NF-κB pathway and a downregulation of the transcriptional effects induced by NPS-1034 (Figure 4A). Moreover, an upregulation of c-caspase-3 and c-caspase-7, and a decrease in surviving and claspin expressions complies with the trend in the apoptotic effects following treatment of TC cell lines at higher NPS-1034 concentrations, verifying the potential apoptotic-effect of NPS-1034 via the TNFR1 pathway (Figure 4B).

## 4. Discussion

This study aimed to evaluate the potential of NPS-1034 in TC cell lines. With a series of examinations, our findings provide evidence for the potential of NPS-1034 in suppressing TC cell survival and confirm its strength in inducing cell death via apoptotic pathways. To date, only three articles on NPS-1034 have been published, and those studies were related to lung cancer and gastric cancer cells [10,16,24]. Our research is the first to evaluate the effect of NPS-1034 on TC in an in vitro study. Confirmation of the therapeutic potential in TC cell lines helps extend the prospect of applying NPS-1034 to different types of cancer.

Our findings comply with the previous result that NPS-1034 may serve as an inhibitor for both Axl and Met receptors [10,16,24]. Additionally, this study further implicated the potential involvement of the TNFR1/NF-κB pathway following NPS-1034 treatment in TC cell lines. TNFR1 regulates a series of downstream pathways, including inflammation, apoptosis, and survival via the NF-κB pathway, which is involved in the expression of antiapoptotic genes, including cIAP1 and FLIPL [25,26]. The inhibitory effect of NPS-1034 on proteins involved in the NF-κB pathway was shown in the downregulation of phosphorylated IKBα, transcriptional factors p50 and p65, and a lowered expression of cIAP1 in both cell lines. However, alterations in the expression of FLIPL could barely be observed in NTERA2 in our data, suggesting that NCCIT and NTERA2 are different in nature. TNFR1 pathway is also associated with apoptotic effects. An upregulation in c-caspase-3 and -7 and a reduction in both claspin and survivin can be observed in our findings, implying the potency to induce apoptosis by NPS-1034. Although the evidence in our research suggests the involvement of the TNFR1 pathway induced by NPS-1034 in TC cell lines, the exact pharmacological interaction of NPS-1034 and its downstream pathway still remains unclear. A more discrete analysis on TNFR1 pathway is required to confirm and elaborate on the exact molecular mechanisms involved.

One of the limitations of our study is that only two TC cell lines, NCCIT and NTERA2, were used. Further studies on other TC cell lines are needed to ascertain the potential of NPS-1034 in treating TC. Moreover, identification of a potential apoptotic signal transduction pathway via the proteome array could only detect certain markers. Mechanisms beyond the membrane used for proteome profiling may thus be neglected. A wide spectrum of targets was analyzed with western blotting to detect additional molecular signals to broaden the scope of knowledge on activating downstream pathways, yet further examinations are needed to uncover underlying mechanisms. Lastly, there were no in vivo experiments in our study to analyze the medical and adverse effects of NPS-1034 for TC treatment. Only in vitro assays were performed considering the principles of the 3Rs: replacement, refinement, and reduction.

## 5. Conclusions

Our research is the first to identify the therapeutic role of NPS-1034 in treating TC cancer cell lines. The results do highlight the potential TNFR1 mechanism involved following NPS-1034 treatment. The study should call attention to the prospect of new TC regimens.

## Data Availability

All data analyzed are inluded in this articel and the origunal data are available on request from the corresponding author.
